# Discovery of the human homolog of sex-determining region (SRY) gene in dioecious plants

**DOI:** 10.1016/j.sjbs.2022.103548

**Published:** 2022-12-23

**Authors:** Mohei EL-Din Solliman, Hany S. Elbarbary, Mohammed Ba Abdullah, Tarek Y.S. Kapiel, Mourad A.M. Aboul-Soud, Heba Allah A. Mohasseb

**Affiliations:** a Plant Biotechnology Department, College of Agricultural and Food Sciences, King Faisal University, P.O. Box 400, Al-Ahsa 31982, Saudi Arabia; b Plant Biotechnology Department, National Research Centre, Dokki-Egypt, Cairo, Egypt; c College of Medicine, King Faisal University, P.O. Box 400, Al-Ahsa 31982, Saudi Arabia; d Internal Medicine Departments, Faculty of Medicine, Menoufiya University, Egypt; e Biology Dept., College of Sciences, King Faisal University, P.O. Box 400, Al-Ahsa 31982, Saudi Arabia; f Botany and Microbiology Department, Faculty of Science, Cairo University, Giza 12613, Egypt; g Chair of Medical and Molecular Genetics Research, Department of Clinical Laboratory Sciences, College of Applied Medical Sciences, King Saud University, P.O. Box 10219, Riyadh 11433, Saudi Arabia

**Keywords:** Dioecious crops, SRY gene, Sex determination, Molecular breeding, Jojoba, Date palm, Pistachios

## Abstract

Sex determination in the early developmental stages of dioecious crops is economically-beneficial. During this study, a human homology of *SRY* gene was successfully identified in dioecious crops. *SRY* gene sequences of date palm and jojoba were submitted to GenBank under the accession numbers KC577225 and MK991776, respectively. This is the first report regarding the novel sex-determination methodology of four dioecious plants (jojoba, date palm, papaya, and pistachios). *SRY* sex gene was found in all the tested dioecious plant and human samples. This novel approach is simple and of significant importance for breeders. It facilitates the unambiguous selection of jojoba and date palm female plants at an early age and reduces the plantation cost of cultivating non-productive male plants. This is a rapid sex-determination technique for dioecious plants and mammals at an early stage. This technique specifically targets the SRY sequence that has been comprehensively investigated in humans. The kit development for the SRY-based sex determination of various crops is in progress.

## 1 Introduction

Sexual dimorphism related dioecism has always been challenging for cultivating trees and crops. Sex identification in dioecious plants is quite complicated particularly at early developmental stages and before flowering. Sex chromosomes are known to separate the sexes in dioecious plants and humans and determine the sexual features of an organism. Traditionally, the symbol ♂ designates male whereas the emblem ♀ designates female. Chromosomes and genes collectively govern sex expression. The unisexual animals contain two types of chromosomes known as autosomes and allosomes. The numbers and morphology of autosomes remain the same in females and males. Contrarily, the numbers and morphology of allosomes (sex chromosomes) differ in females and males and they carry sex-determination genes. Two sex chromosomes are found in unisexual diploid individuals whereas all the remaining chromosomes are autosomes (Solliman et al., 2019). Sex chromosomes in some fish species could be similar and small-sized. Sex chromosomes in other plants are heteromorphic, which are similar to humans, drosophila, or neosex chromosomes (Liu et al., 2004). The flowers of most plants and long-lived dioecious species contain both reproductive organs (male and female) and are known as hermaphrodite or bisexual. Similarly, the individuals of earthworms and nematodes possess both reproductive organs (male and female). The reproductive organs of monoecious plants (coconut, castor, maize) are found in different flowers of the same plant. However, dioecious species (papaya, date palm, and jojoba) contain female and male flowers on separate plants. The physical separation between female and male plants is known as sex determination. Multiple reviews have documented the sex determination mechanisms in various crops (Charlesworth, 1996). Sex identification in dioecious plants is usually difficult at early developmental stage and before flowering. Male and female flower identification in fruit and seed-producing plants is crucial from the breeder's perspective. The plants in which female flower representation is more important than male flowers include *Actinidia deliciosa* (Shirkot et al., 2002); *Phoenix dactylifera* (Solliman et al., 2019); *Carica papaya* (Parasnis et al., 2000); *Borassus flabellifer* (George and Karun, 2011); *Hippophae rhamnoides* (Persson and Nybom, 1998, Sharma et al., 2010); *Myristica fragrans* (Shibu et al., 2000); *Piper longum* (Manoj et al., 2008); *Pistacia vera* (Hormaza et al., 1994, Hormaza and Wunsch, 2007), and *Simmondsia chinensis* (Agarwal et al., 2008, Agarwal et al., 2011, Agrawal et al., 2007, Mohasseb et al., 2009). To achievebetter date palm production, female plants should be more than males. However, it is currently impossible to determine plant sex at the early developmental stages and it can only be identified on blooming (at approximately-five years of age). Previously, many attempts have been made to determine plant sex at an early stage. However, most of the efforts were not successful and led to incalculable female plant field population and production. The long dioecism and juvenility phases make date palm breeding a challenging task. These characteristics necessitate the date palm sex determination in the early developmental stages to achieve the correct plantation ratio in the field (Solliman et al., 2019).

### 1.1 Xx-XY system of chromosomal sex determinations

The females contain only one type of egg (XX) because of the meiosis during gamete formation whereas the males produce two types of sperms (XY, 50% of each chromosome). During the fertilization, if the sperm containing X-chromosomes fuses with the egg, then the zygote develops into a female (XX). Contrarily, if the sperm containing Y-chromosomes fuses with the egg, then the zygote develops into a male (XY). Homogametic female contains only one type of gamete (X-bearing) (Cherif et al., 2016) whereas the heterogametic male contains two types of gametes (X- and Y-bearing). At fertilization, the sex ratio is expected to be 1:1 as males equally produce X- and Y-bearing sperms. Sex determination in various organisms is represented by the female genotype/ male genotype such as drosophila (XX XY); birds, fish, and butterflies (WZ WW); and humans and other mammals (XX XY). XX/XY is the most common sex-determination system in mammals and humans. *SRY* gene present on the Y chromosome produces a male organism. Humans also possess a similar *SRY* gene. Autosomes might contain the gene for transforming the normal male (XY) into a female. Similar impacts of a single recessive gene have also been observed in humans and some animal species (dogs, pigs, goats, and other long-lived species). The mixed pollination in jojoba has sex determination difficult before flowering (Roussos et al., 1999). The seed production of jojoba is high (700–800 gm) and it is characterized by a high ratio of active secondary compounds that are utilized in various industries. Jojoba could be successfully cultivated in the desert regions of Arab countries (Shehata et al., 2018).

### 1.2 Sex-linked markers in dioecious plants

Sex determination is crucial for estimating the proportions of female and male individuals in the field and studying the impact of sex distribution-related factors. Sex determination is particularly of economic importance as the sexual phenotypes could identify the gender of reproductively inactive dioecious plants, humans, and other mammals (Manoj et al., 2008, McKeown et al., 2000, McLetchie and Collins, 2001, Michael and Brauner, 2004). The lack of reliable morphological methods has driven perpetual attempts to determine the sex of dioecious crops at early developmental stages, which have failed so far. These attempts only lead to an unpredictable female plant population and uncertain crop production in the field.

Polymerase chain reaction (PCR) has been employed for isolating a human and animal homolog *SRY* gene in plants with a conserved motif. Sex chromosomes of several plants contain sex-determining genes within a tiny recombination-free region (blue; only 10% of chromosome 1 of papaya) (Liu et al., 2004). The current work presents a reliable advanced molecular technique to efficiently differentiate among productive female dioecious and only pollens producing male plants. This technical advancement for the sex identification of dioecious plants is based on PCR-based molecular approaches employed in humans and forensic medicine (Parasnis et al., 2000, Steinlechner et al., 2002, Sullivan et al., 1993, Thangaraj et al., 2002). Previously, we have developed a rapid PCR-based method for *SRY* gene detection in dioecious plants (Solliman et al., 2019). Satellite sequences within higher-order repeats on human chromosomes (X and Y) were PCR-amplified using genomic DNA from bone, blood, and other forensic samples. The co-amplification of X and Y sequences was also carried out by employing PCR (Solliman et al., 2019). Several PCR-based genomes walking strategies entailing combined vectorette and suppression PCR walking have been reported (Reddy et al., 2002, Rosenthal and Jones, 1990).

This study establishes the presence of SRY-related sequences in dioecious plants. The PCR-based strategy was employed for the isolation of the *SRY* gene possessing homology to the *SRY* gene’s conserved motif of humans and other mammals.

## 2 Materials and methods

### 2.1 Plant DNA isolation

DNA isolation from plant samples was carried out by following our previously reported protocol (Solliman et al., 2019).

### 2.2 Polymerase chain reaction (PCR)

SRY gene-walking strategy and PCR conditions were adjusted based on our previously reported protocol on papaya and protocols mentioned in other studies (Solliman et al., 2019, Mohasseb et al., 2020, Dominguez and Lopez-Larrea, 1994). Mastermix containing DreamTaq DNA Polymerase (Thermo Scientific) was prepared by following the instructions of the manufacturer.

### 2.3 Sex identification in early dioecious plant stages

Dioecious plant samples were randomly collected from King Faisal University Research and Training Station and DNA was isolated (Solliman et al., 2019, Lo et al., 1998). Human samples (female and male DNA) were acquired from the College of Medicine, KFU, Saudi Arabia. Primer 3 program (https://primer3.ut.ee/) facilitated the targeting of male-specific SRY marker (located on the Y chromosome sex-determination region) in the oligo primers employed in the study (Table 1)***.***

**Table 1:** Primer pairs used in PCR to analyze the amplification of a Dioecious plant male-specific SRY gene situated in the Y chromosome sex determination region was designed as forward (F) and reverse(R) primer as follows.

Primer’s name	sequences	Design as	Tm	Product length	Combination
SR DP F1	5-GACAGCGCTGTGCAAAGA-3	Forward (F) F1	59.97	409	A
SR DP R1	5′-GGCATACGAGAAGCTGGGAT-3′	Reverse (R) R1	59.97		
SR DP F2	5-AACTCTAGAAATGCTGAAACA-3	Forward (F) F2	59.77	226	B
SR DP R2	5-TACCTGCAAGTACAG 3	REVERSE (R) R2	60.11		
SR DP F3	5′-TCAGTCCAGACGTGCAAAG-3′	Forward (F) F3	60.25	244	C
SR DP R3	5′-ATCCTGGATATCCCTGCTCT-3′.	Reverse (R) R3	59.75		

SRY primers flanked a region of 300 to 550 bp (Solliman et al., 2019). Gel electrophoresis of PCR samples was carried out to separate and detect the *SRY* gene.

### 2.4 SRY gene isolation

Genomic DNA isolated from Jojoba adult shrub was subjected to overnight restriction-digestion at 37 °C. *Bam*HI and *Bgl*II *Sou3*AI (NEB) enzymes were mainly used whereas oligonucleotides included: ADOP-32 5′-AATACGACTCACTATAGGGCGGCCGCCCGGGC-3′ and ADOP-27 5′-CACTATACCCGCCGGCGGGCCCGCT-3. Both oligonucleotides were ordered from Macrogen Inc. (South Korea).

#### 2.4.1 PCR amplification (primary)

PCR amplification was carried out using a thermal cycler (Applied Biosystems Veriti®). Adaptor primer T7 (5′-AATACGACTCACTATAGGGC-3′) and gene-specific SRY-2 primer (Jojo-SRY) were employed to amplify the target gene. SRY-1R and SRY_2R primer sequences were as 5′- GGGCTGTAAGTTATCGTAAAAGGAGC-3′ and 5′-CCTAGCTGGTC ACGTTGACCTTTTGTCC-3′, respectively.

#### 2.4.2 PCR amplification (secondary)

PCR sample from primary amplification served as a template after purification through phenol extraction. DNA precipitates were resuspended in TE (20 µl). Different dilutions of the excised band were prepared and subjected to PCR amplification by employing SRY-2R and T7 primers. SRY-2 primer sequence consisted of 5′-GGAGCATCTAGGTAGGTCTTTGTAGCC-3′.

### 2.5 DNA Sanger sequencing

PCR amplicons were subjected to Sanger sequencing according to Macrogen Inc. (South Korea).

### 2.6 Bioinformatics

Genbank database and CLCVector program were used to analyze the obtained DNA sequence. Homology searches were performed through FASTA whereas multiple sequence alignment was carried out using CLUSTALW.

## 3 Results

The study aimed to elaborate the *SRY* gene (sex-determining gene) in plants. *SRY* gene was isolated from dioecious plants and humans. We have first time reported *SRY*-related gene sequence in dioecious plants. PCR identification of human homolog *SRY* gene with conserved motif was carried out. *SRY* gene-based sex determination in dioecious plants, humans, and other mammals involved the amplification of DNA segments. This technique aimed to find sex determination-related markers in the dioecious plants (Date palm, jojoba, papaya, *Silene latifolia*, and Pistachios). Different PCR reactions deduced the plant sex as male using primers of other Y chromosome-specific markers (Universal and SRY primers). SRY marker has already yielded a PCR product of 360 bp overlapping some previously published human and dioecious plant sequences. Therefore, it is necessary to conduct additional PCR for establishing the plant gender. The male sample amplification generated only one band (300–360 bp), which was putatively identified as 360 bp SRY. The identification of unknown DNA sequences flanking known regions is crucial for analyzing the gene sequence. Multiple PCR protocols have been adopted for isolating the novel DNA promoters adjacent to already known DNA sequences and for cDNAs (Solliman, 2002, Hui et al., 1998). Dioecious Plant sex determination at early stages has been attempted by several researchers without considerable success. We studied the sequences between SRY forward and reverse primers to achieve a smaller PCR product-producing primer in various regions of humans, papaya, date palm, and jojoba. During the study, a previously published SRY-1R primer (reverse primer) was selected (Solliman et al., 2019) whereas ADOPTER sequences served as forward primer (Solliman et al., 2019, Triglia et al., 1988). A primer pair consisted of a universal forward primer SRY_uF and reverse primer SRY_uR. SRY-uF forward primer sequence was as: SRY_F 5′-AGAAGTGAGTTTTGGATAGTAAAATAAGTTTCGA-3′ whereas SRY-uR reverse primer sequence was as: 5′-CTCACCGCAGCAACGGGACCGCTACAGCCACTGG-3′.

**Fig. 1:**
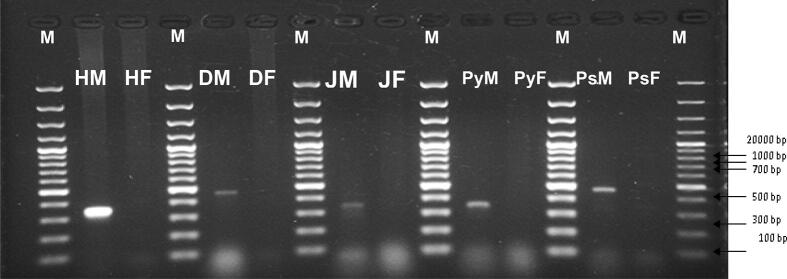
PCR amplification of SRY gene in human males and females (HM(335 bp) and HF(00 bp)) in comparison to dioecious plants (dates: (DM(500 bp) and DF(00 bp)), jojoba: (JM and JF), papaya: (PyM(335 bp) and PyF(00 bp)), and Pistachios: (PsM(335 bp) and PsF(00 bp)) using specific primers (universal SryF + universal SryR). Lane M represents the DNA marker (100 bp).

The results revealed that initially female and male amplicons become easy to be distinguishable. PCR products depicted unique DNA fragments related to only male plant samples (Fig. 1). These DNA fragments, absent in female plant samples, were subjected to further investigations.

**Fig. 2:**
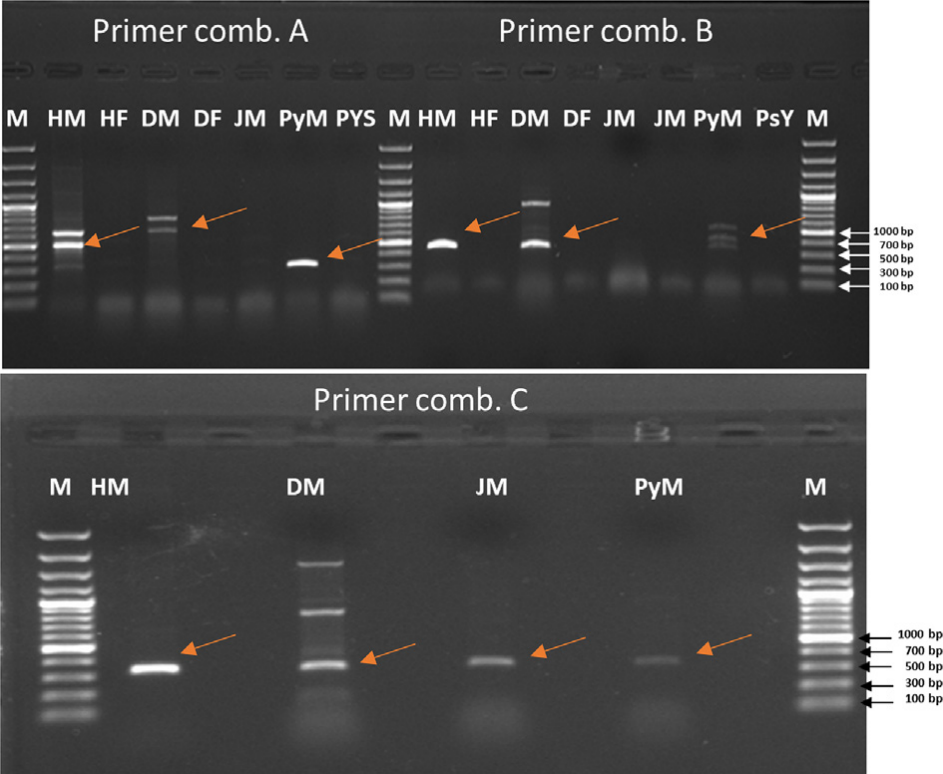
PCR amplification of SRY gene with different primer combinations. **Primer combination A**, PCR amplification of human male and female (HM and HF) in comparison to dioecious plants (dates (male, DM), jojoba (male, JM), papaya (male, PyM), and Pistachios (male, PsM)) using specific primers (SryF1 + SryR1). Lane M represents the DNA marker (100 bp). **Primer combination B**, PCR amplification of human male and female (HM and HF) in comparison to dioecious plants (dates (male and female), jojoba (male and female), papaya (papaya male) and pistachios (Pistachios male)) using specific primers (universal SryF2 + universal SryR2). **Primer combination C**, PCR amplification of human male and female (HM and HF) in comparison to dioecious plants (dates (male), jojoba (male), papaya (male), and Pistachios (male)) using specific primers (SryF3 + SryR3). Lane M represents the DNA marker (100 bp). Abbreviations, Human male (HM), Human Female (HF), Dates male (DM), Jojoba male (JM), Papaya male (PyM), and Pistachios male (PsM).

This study developed a new method for early sex diagnosis in the jojoba plant by employing the male-specific *SRY* gene (sex-determining region Y) as a standard sex marker. The first pair of gene-specific primers amplified a fragment of approximately 550 bp (Fig. 2, Comb. A) whereas the second pair of primers generated a product of 700 bp (Fig. 2, Comb. B) in dioecious male plants. These fragments were absent in female dioecious plants (Fig. 2). Two types of gene isolation strategies by new PCR-walking were optimized to differentiate among *SRY* gene sequences of four dioecious male and female plants. This study demonstrates molecular methods for the sex determination (male and female) in dioecious cultivars (date palm, jojoba, papaya, and pistachios) (Fig. 2). One or two non-SRY specific bands were also obtained with DM (Comb. A) and PyM (Comb. B). However, the human male-specific SYR amplicon band (absent in female cultivars) was always present in the male cultivar thus indicating its sex specificity. Notably, single human SRY-specific PCR bands were successfully obtained from data palm, papaya, and pistachios without any non-specific amplification. These results established the effectiveness of the applied thermal program (Fig. 2, Comb. C).

**Fig. 3:**
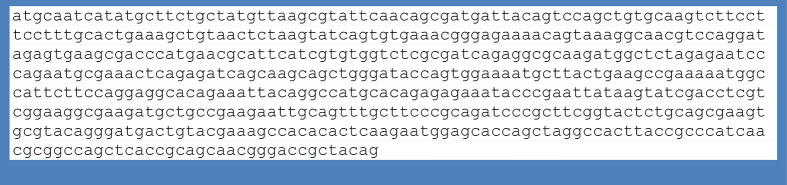
Sequence submitted to Genbank (accession no. BankIt1598036 DPSRY1 KC577225).

**Fig. 4:**
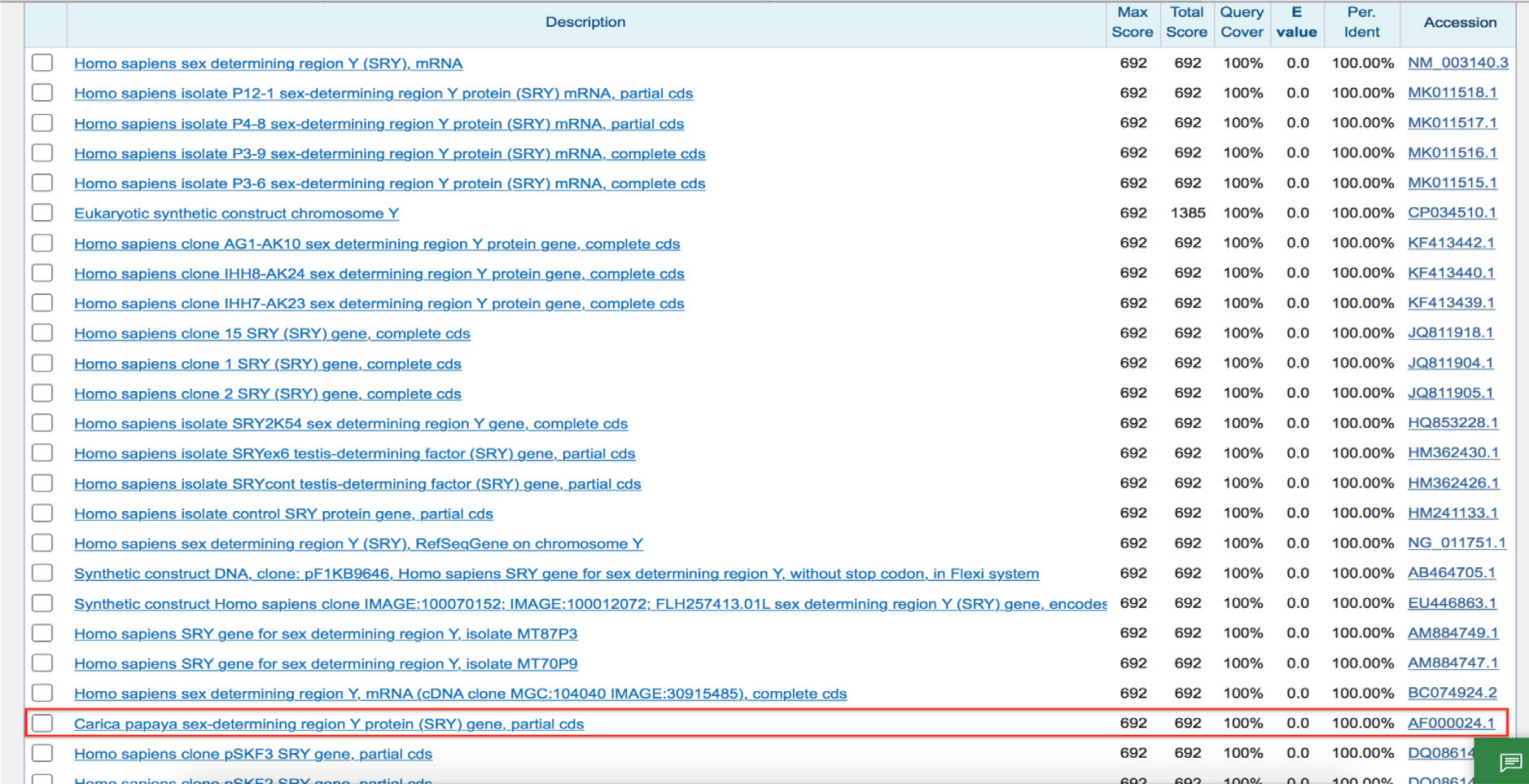

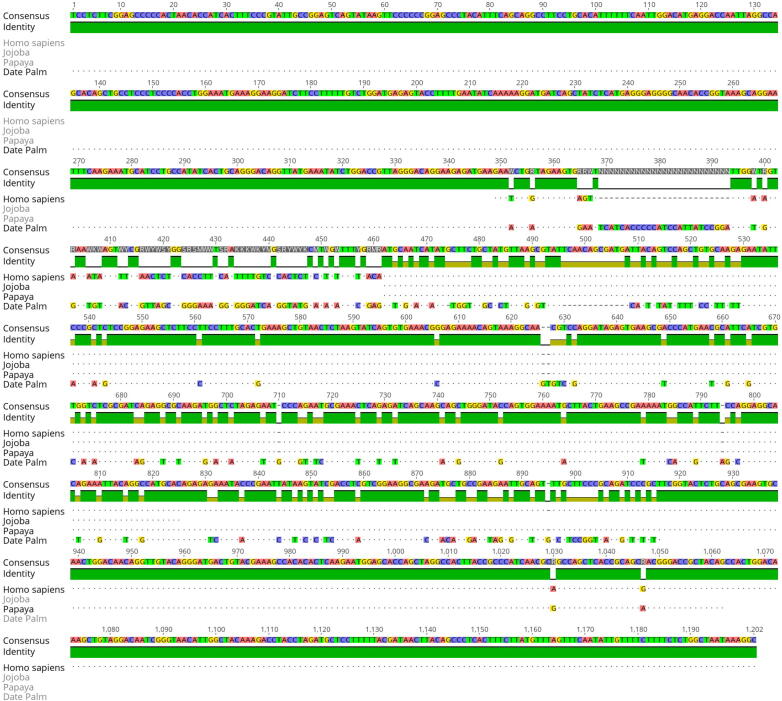
(a) sry sequence alignment presents identical sequences of control (male) and five cases corresponding to sry sequence from plants and humans. (b) Sequence alignment of sry gene of human (jq811918.1), jojoba (mk991776.1), papaya (af000024.1), and date palm (kc577225.1). The consensus sequence is on the top. the green bar indicates the similarity to the consensus. Identical nucleotides are shown in dots.

**Fig. 5:**
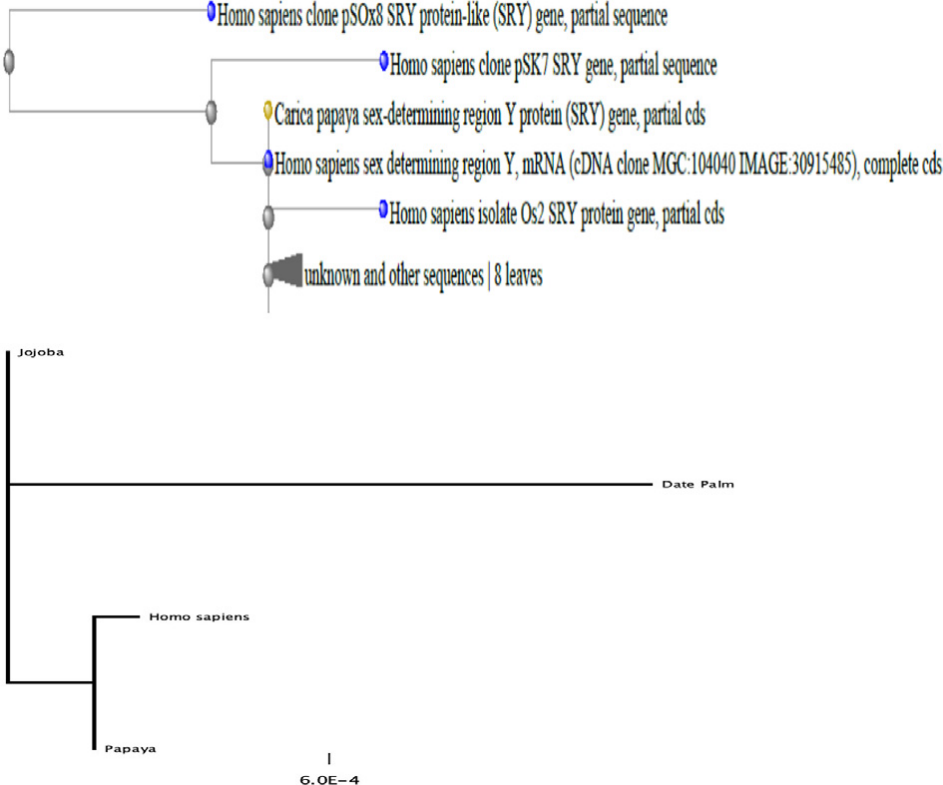
Phylogenetic relationships of jojoba, *Carica papaya*, and *Homo sapiens* sex-determining region Y. Phylogenetic analysis of proteins (SRY protein and sex-determining region Y protein) were separately performed using reported members of Homo sapiens sex-determining region Y and *Carica papaya*. The neighbor-joining method was followed to construct the phylogenetic tree from full-length amino acid sequences at 100 bootstrap values. Phylogenetic analysis of SRY gene of Humans (JQ811918.1), Jojoba (MK991776.1), Papaya (AF000024.1), and Date palm (KC577225.1). The unrooted phylogenetic trees were constructed by the Neighbour-joining method. The scale bar represents the tree distance corresponding to 0.006 nucleotide substitution/kb.

PCR amplification of the *SRY* gene was carried out using the genomic DNA of dioecious plants. Fig. 2 depicts some differences in the DNA size that were also confirmed through *SRY* gene sequencing and sequence alignment (Fig. 5). Sequence alignments presented 100% identical sequences of control (male) and five cases corresponding to SRY sequences from plants and humans (Fig. 3, Fig. 4, Fig. 5).

Subsequently, the ExPASy translate tool was used for translating nucleotide sequences into polypeptides, and the BLASTP algorithm was employed for the identity search. Both SRY-gene analogs were highly similar to *Homo sapiens* sequence (GenBank accession number: gb|JQ811918.1) and papaya *SRY* gene sequence (GenBank accession number: gb|AF000024.1|CPAF000024) (Fig. 4, Fig. 5).

Phylogenetic analyses of SRY nucleotide sequences and genomic flanks in humans and plants were conducted. The phylogenetic tree was constructed using the SRY coding region (360 bp) in humans and plants. A maximum-likelihood tree was derived through the heuristic search by following the tree-bisection-reconnection branch-swapping method and the results of the distance tree (https://www.ncbi.nlm.nih.gov/blast/treeview/). SRY coding region (360 bp) and translated amino acids-based phylogenetic tree exhibited higher similarity with nucleotide phylogeny (Fig. 5).

## 4 Discussion

A mixed population of male and female plants is necessary for successful fruit-bearing. The presence of one male plant among six female plants is necessary. Multiple reviews have been published regarding the evolution of sex chromosomes (Charlesworth, 1996, Charlesworth, 2002). Sexual dimorphism associated with dioecism has always been challenging for plant cultivation, particularly in the case of woody trees. Sex identification in dioecious plants is difficult, specifically before reaching to flowering stage and during early developmental stages. Date palm plant sex could be identified only at the blooming stage (at approximately-five years of age). Therefore, acquiring a proper male-to-female plant ratio in the field is impossible, which significantly reduces date production. This situation urges the plant sex identification during the early developmental stages to achieve a correct plantation ratio. Several attempts have been made for plant sex identification in the early stages. However, they could not produce successful results and led to an unpredictable female plant population in the field thus causing uncertain crop production (Agrawal et al., 2007, Agarwal et al., 2011, Agarwal et al., 2008, Mohasseb et al., 2009, Matsunaga and Kawano, 2001, Scutt et al., 2002, Santos et al., 1998, Triglia et al., 1988, Solliman et al., 2020). This study deduced that the human genetic code shares genes with other animal and plant species. According to our previous study, such widespread gene transfer among species is quite common in animals and plants (Solliman et al., 2019). The sex-determining function of the *SRY* gene is an excellent example of this phenomenon. DNA sequence similarity could trace them back to a single ancestor *SRY* gene. Therefore, the *SRY* gene is considered a member of the same family in dioecious plants, humans, and animals. Furthermore, we are focusing to develop sex***-***determination PCR kits at King Faisal University, Saudi Arabia. These kits will use PCR primers for conducting mass *SRY* gene-based molecular screening to determine the sex of dioecious plants.

## 5 Conclusion

This study offers a breakthrough in the sex determination of dioecious plants including date palm, jojoba, and pistachios. Nested PCR was conducted to amplify and identify the partial sequences related to the sex determination region at the Y chromosome (SRY). Dioecy-*SRY* genes shared higher similarities with humans and papaya. Primers (two sets) amplified the fragments of size 330 bp and 470 bp, respectively, in human male and dioecious male plant samples (Fig. 1). Dioecy-SRY_F and dioecy-SRY_R primers sequences were as *5*′*-cggccctctaagtatctgtgcgcaacg-3*′ and *5*′*-gtttgcacttcgaagcagag-3*′, respectively.

## 6 Novelty statement

The current study was conducted to develop a simplex PCR-based procedure that is reproducible and applicable for early verification of sex in dioecious male plants, which is designed for the first time based on the human SRY gene homolog. This protocol was optimized for early sex verification of dioecious male plants before flowering and during the extremely early developmental phases. This discovery is envisaged to have significant beneficial economic implications for breeders, especially before the propagation of flowering dioecious plants.

## Declaration of Competing Interest

The authors declare that they have no known competing financial interests or personal relationships that could have appeared to influence the work reported in this paper.

## References

[b0010] Agarwal M., Shrivastava N., Padh H. (2008). Advances in molecular marker techniques and their applications in plant sciences. Plant Cell Rep..

[b0015] Agarwal M., Shrivastava N., Padh H. (2011). Development of sex-linked AFLP markers in Simmondsia chinensis. Plant Breed..

[b0020] Agrawal V., Sharma K., Gupta S., Kumar R., Prasad M. (2007). Identification of sex in *Simmondsia chinensis* (jojoba) using RAPD markers. Plant Biotechnol. Rep..

[b0025] Charlesworth B. (1996). The evolution of chromosomal sex determination and dosage compensation. Curr. Biol..

[b0030] Charlesworth, B., 2002. The evolution of chromosomal sex determination. In: Short R (Ed.), The Genetics and Biology of Sex Determination. John Wiley: Chichester, UK. pp. 207–219.

[b0035] Cherif E., Zehdi-Azouzi S., Crabos A., Castillo K., Chabrillange N., Pintaud J.C. (2016). Evolution of sex chromosomes prior to speciation in the dioecious Phoenix species. J. Evol. Biol..

[b0040] Dominguez O., Lopez-Larrea C. (1994). Gene walking by unpredictably primed PCR. Nucleic Acids Res.

[b0045] George J., Karun A. (2011). Marker assisted detection of seed sex ratio in palmyrah palm (*Borassus flabellifer* L.). Curr. Sci..

[b0050] Hormaza J.I., Dollo L., Polito V.S. (1994). Determination of relatedness and geographical movements of *Pistacia vera* (Pistachio; Anacardiaceae) germplasm by RAPD analysis. Econ. Bot..

[b0055] Hormaza J.I., Wunsch A., Kole C. (2007).

[b9005] Hui E.K., Wang P.C., Lo S.J. (1998). Strategies for cloning unknown cellular flanking DNA sequences from foreign integrants. Cell Mol. Life Sci..

[b0065] Liu Z., Moore P.H., Ma H., Ackerman C.M., Ragiba M., Pearl H.M. (2004). A primitive Y chromosome in papaya marks the beginning of sex chromosome evolution. Nature.

[b0070] Lo Y.M.D., Tein M.S.C., Lau T.K., Haines C.J., Leung T.N., Poon P.M.K. (1998). Quantitative analysis of fetal DNA in maternal plasma and serum: implication for noninvasive prenatal diagnosis. Am. J. Hum. Genet..

[b0075] Manoj P., Banerjee N.S., Ravichandran P. (2008). Development of sex specific molecular markers in dioecious *Piper longum* L. plants by differential display. JATIT.

[b0080] Matsunaga S., Kawano S. (2001). Sex determination by sex chromosomes in dioecious plants. Plant Biol..

[b0085] McKeown B., Sickley J., Riordan A. (2000). Gender assignment by PCR of the SRY gene: an improvement on amelogenin. Prog. Forensic Genet..

[b0090] McLetchie D.N., Collins A.L. (2001). Identification of DNA regions specific to the X and Y chromosomes in Sphaerocarpos texanus. Bryologist.

[b0095] Michael A., Brauner P. (2004). Erroneous gender identification by amelogenin sex test. J. Forensic Sci..

[b0100] Mohasseb A.H.-A., Solliman M.E.-D., Al-Mssallem I.S., Abdullah M.B., Shehata W.F., El-Shemy H.E. (2020). Salt tolerant phenomena, sequencing and characterization of a glyoxalase I (Jojo-Gly I) gene from Jojoba in comparison with other glyoxalase I genes. Plants.

[b0105] Mohasseb H.A., Moursy H.A., El-Bahr M.K. (2009). Sex determination of jojoba using RAPD markers and sry gene primer combined with RAPD primers. Res. J. Cell Mol. Biol..

[b0110] Parasnis A.S., Gupta V.S., Tamhankar S.A., Ranjekar P.K. (2000). A highly reliable sex diagnostic PCR assay for mass screening of papaya seedlings. Mol. Breeding.

[b0115] Persson H.A., Nybom H. (1998). Genetic sex determination and RAPD marker segregation in dioecious species sea buckthorn (*Hippophae rhamnoides* L.). Hereditas Landskrona.

[b0120] Reddy M.K., Nair S., Sopory S.K. (2002). A new approach for efficient directional genome walking using Polymerase Chain Reaction. Anal. Biochem..

[b0125] Rosenthal A., Jones D.S. (1990). Genomic walking and sequencing by oligo-cassette mediated polymerase chain reaction. Nucleic Acids Res.

[b0130] Roussos P.A., Tolia-Marioli A., Pontikis C.A., Kotsias D. (1999). Rapid multiplication of Jojoba seedlings by in vitro culture. Plant Cell Tissue Organ Cult..

[b0005] Santos F.R., Pandya A., Tyler-Smith C. (1998). Reliability of DNA-based sex tests. Nat. Genet..

[b0135] Scutt C.P., Jenkins T., Furuya M., Gilmartin P.M. (2002). Male specific genes from dioecious white campion identified by fluorescent differential display. Plant Cell Physiol..

[b0140] Sharma A., Zinta G., Rana S., Shirkot P. (2010). Molecular identification of sex in *Hippophae rhamnoides* L. using isozyme and RAPD markers. Forestry Stud. China.

[b0145] Shehata W.F., Solliman M.M., Mohasseb H.A., Al-Khateeb A.A., Aldaej M.I., Alturki S.M. (2018). Protocol of *in vitro* Jojoba (*Simmondsia chinensis* (Link) Schneider) Callus Induction. Pak. J. Biol. Sci..

[b0150] Shibu M.P., Ravishankar K.V., Anand L., Ganeshaiah K.N., Shaanker U. (2000). Identification of sex- specific DNA markers in the dioecious tree, nutmeg (*Myristica fragrans* Houtt.). PGR Newsletter.

[b0155] Shirkot P., Sharma D.R., Mohapatra T. (2002). Molecular identification of sex in *Actinidia deliciosa* var. deliciosa by RAPD markers. Sci. Hortic..

[b9000] Solliman mohei EL-Din. Isolation and Characterization of Plant Promoters for High Level and Tissue-Specific Expression of Foreign Genes in Transgenic Plants. Ph.D. from ICGEB (INTERNATIONAL CENTER FOR GENETIC ENGINEERING AND BIOTECHNOLOGY, 1998–2002), INDIA.

[b0160] Solliman M.-E.-M., Mohasseb H.A., Al-Khateeb A.A., Al-Khateeb S.A., Chowdhury K., El-Shemy H.A., Aldaej M.I. (2019). Identification and Sequencing of Date-SRY Gene: A Novel Tool for Sex Determination of Date Palm (Phoenix dactylifera L.). Saudi J. Biol. Sci..

[b0165] Solliman M.M.E.-D., Mohasseb H.A.A., Al-Khateeb A.A., Al-Khateeb S.A., Shehata W.F. (2020). Induce of new tomato varieties resistance to fungal using Agrobacterium-mediated Transformation. Fresenius Environ. Bull..

[b0170] Steinlechner M., Berger B., Niederstatter H., Parson W. (2002). Rare failures in the amelogenin sex test. Int. J. Leg. Med..

[b0175] Sullivan K.M., Mannucci A., Kimpton C.P., Gill P. (1993). A rapid and quantitative DNA sex test: fluorescence-based PCR analysis X-Y homologous gene amelogenin. BioTechniques.

[b0180] Thangaraj K., Reddy A.G., Singh L. (2002). Is the amelogenin reliable for gender identification in forensic casework and prenatal diagnosis?. Int. J. Leg. Med..

[b0185] Triglia T., Peterson M.G., Kemp D.J. (1988). A procedure for in vitro amplification of DNA segments that lie outside the boundaries of known sequences. Nucleic Acids Res.

